# Research inequity in the plant sciences

**DOI:** 10.1002/aps3.11417

**Published:** 2021-04-30

**Authors:** Theresa M. Culley, Megan Philpott, Robert Tunison, Benjamin J. Merritt, José M. Barreiro Sanchez, Alexis Wafer, Rebecca Holdren

**Affiliations:** ^1^ Department of Biological Sciences University of Cincinnati 614 Rieveschl Hall Cincinnati Ohio 45221‐0006 USA; ^2^ Center for Conservation and Research of Endangered Wildlife Cincinnati Zoo and Botanical Garden 3400 Vine Street Cincinnati Ohio 45220 USA; ^3^ College of Forest Resources and Environmental Science Michigan Technological University 1400 Townsend Drive Houghton Michigan 49931 USA; ^4^ Department of Evolution, Ecology and Organismal Biology The Ohio State University 318 W. 12th Avenue Columbus Ohio 43210 USA

**Keywords:** disparity, equity, inclusivity

## Abstract

Do all plant biologists worldwide have equal access to novel methods, enabling them to be equally productive, publish, and receive credit for their research? Or does reduced access to cutting‐edge techniques in countries with lower financial resources create an inequity for researchers located there? Such disparities and biases do exist within our discipline and must be addressed if we are to move forward as a more just society. *Applications in Plant Sciences* has taken steps to address this important issue of research inequity, as outlined below. We now call upon the entire botanical community—researchers, editors and reviewers, funding agencies, and publishers—to work together toward a more equitable environment for all researchers around the world.

As people around the world confronted social inequities during this past year with racial unrest, political strife, and an international pandemic, plant biologists should also reflect upon biases and disparities within our own field. Inequity based on gender and race/ethnicity is a long‐standing problem recognized within the sciences (e.g., Moss‐Racusin et al., 2012; West et al., 2013; Odekunle, 2020; Oreskes, 2020), which undoubtedly still persists today. There is also disparity associated with language—researchers who do not have English as a first language often describe difficulties in publishing their work in English‐language journals (Amano et al., 2016; Pérez Ortega, 2020; Ramírez‐Castañeda, 2020), often opting to publish elsewhere. But are there also other ways inequity may exist within the plant sciences on a more global scale? Do all researchers have equal access to the resources they need to contribute to our profession? Furthermore, are all researchers given the proper recognition they deserve for their accomplishments, regardless of their institution, country, or socioeconomic status? These are important questions to consider as plant biologists increasingly join across political and geographical boundaries to tackle crucial problems such as climate change, habitat modification, and species extinction.

Solving these complex problems often requires the development and implementation of new tools and methods. Unfortunately, cutting‐edge methods are often prohibitively expensive when first introduced and may only be within the reach of well‐funded laboratories located in more wealthy countries (e.g., as defined by the International Monetary Fund, 2020). This is particularly true for genetic and molecular techniques, such as second‐ and third‐generation sequencing methods and CRISPR/Cas9 technology. As the cost of a particular method declines over time, researchers with limited funding can eventually afford to incorporate it into their own work. Consequently, a distinct pattern of a bell‐shaped distribution may emerge when looking at a given method published over time (Fig. 1). The few initial adopters may be from well‐funded laboratories in financially secure countries, followed over time by an ever‐increasing mix of researchers from all over the world, and ending with researchers located in countries with more limited means or associated with smaller institutions who can only later afford the technology. This pattern creates a disparity in which well‐funded laboratories in wealthier countries have the option of shifting from one novel technique to another on the front of subsequent method curves. In contrast, other researchers may be relegated to lagging at the back of each curve simply because they cannot access or afford new technologies. This “technology treadmill” also occurs in agriculture when new, expensive practices are incorporated into farms (Levins and Cochrane, 1996).

**FIGURE 1:**
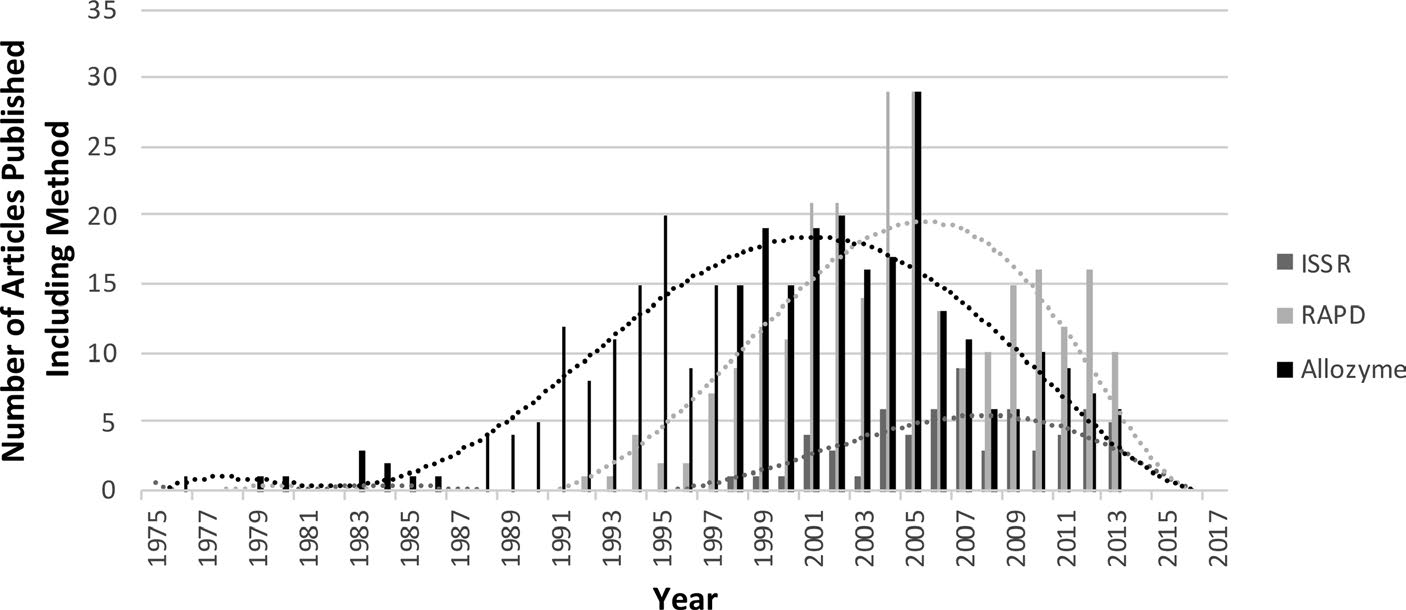
Inclusion of a specific genetic technique in the published literature typically follows a bell‐shaped curve over time. The graph shows the number of published articles that mention allozyme markers (black bars; 390 articles), RAPD markers (light gray bars; 273 articles), and ISSRs (dark gray bars; 63 articles) in the JSTOR PLANTS database, which represents largely English‐only journals worldwide. The full text of articles published up to 2017 were searched in the journal categories of “Botany and Plant Sciences” and “Biological Sciences” using the terms “RAPD” or “allozyme” in plant species.

There is also another unfortunate outcome to this situation. Any method over time will eventually be considered too outdated by many higher‐impact peer‐reviewed journals, ostensibly because the method is not “current enough” to fulfill their criteria of innovative research. Consequently, researchers of more limited means who are using methods perceived by others as outdated may find themselves unable to publish their work in higher‐profile journals; this is especially problematic when publication in international journals may be key to their career advancement. Reduced opportunities to publish may occur on top of language difficulties that complicate the issue even further (Huttner‐Koros, 2015; Woolston and Osório, 2019; Pérez Ortega, 2020), particularly if some reviewers and editors discriminate against researchers from primarily non‐English speaking countries (e.g., Romero‐Olivares, 2019; Ramírez‐Castañeda, 2020). Thus, papers with “older” methods may be more likely to appear in regional journals, often in a non‐English language and not indexed in databases such as the Web of Science, and therefore not be readily accessible to other scientists globally (Amano et al., 2016). The irony is that many researchers are located in botanically rich but economically challenged countries within the tropics—where botanical knowledge may be limited relative to other locations (Amano and Sutherland, 2013; Reboredo Segovia et al., 2020). Thus, their contributions may be incredibly valuable, especially if a given species in that tropical locality has never before been examined. The end result is geographic‐based disparity, driven in part by unequal access to new methods and culminating in some researchers being unable to publish their work in international journals because their methods are viewed as antiquated, even though the rest of the content may be valuable to a global audience. In the end, the entire community loses out.

What can be done to erase this inequity? One approach is to promote greater collaboration between more financially privileged researchers and those investigators lacking resources necessary to carry out the research, but who have deep knowledge of the plant species themselves. There are situations where this arrangement has certainly worked well, but it comes with a potential danger of a perceived imbalance in the relationship. Specifically, it is critical that researchers in less affluent countries be adequately recognized for their work as full partners and coauthors on papers, rather than being used simply for access to the study species and only mentioned in the acknowledgements—known as “parachute science” (De Vos, 2020; Stefanoudis et al., 2020). Authorship should acknowledge the contribution of *all* individuals without whom the study could not have been completed (Gunturiz Albarracín et al., 2020). This can be easily achieved using the Contributor Roles Taxonomy (CRediT; https://casrai.org/credit/) to indicate roles played by each contributor to a scientific paper.

An arguably better approach, but much more difficult to achieve, is to work together as a profession to (1) recognize the value of international researchers and their contributions, and (2) empower all plant biologists, regardless of their geographic location, country, and/or socioeconomic status, with equal access to methods that result in publishable papers. For example, scientists in the United States and other privileged countries should make every effort to cite articles by international researchers, invite them to give departmental seminars, and recruit their students into graduate programs and as postdoctoral researchers (and provide continuing support if they then choose to return to their country of origin). Furthermore, many universities and research institutions in tropical countries desperately need access to new technology, given the scarcity of scientific funding in their home country. Collaborating, more privileged researchers should include colleagues in these countries on grant proposals to facilitate research at that site. However, this requires that federal funding agencies allow the transfer of a portion of allocated funds to other countries. At the very least, large funding agencies should encourage the inclusion of local researchers and field guides if research is conducted abroad, with funding allowed for that expense. In addition, alternative methodological solutions could also be pursued that are not as costly, such as those outlined in a special issue of *Applications in Plant Sciences* in April 2020 (Dean et al., 2020).

There are also steps that can be taken within the publication process. Reviewers should think about their own implicit biases and how that might affect their reviews, especially involving authors who do not have English as a first language (Romero‐Olivares, 2019); for example, reviewers may need to focus more on the scientific content rather than on the grammar (Pérez Ortega, 2020). There can also be bias during peer review when authors have Latin‐ or Asian‐sounding names (even though English might be their original language), a situation circumvented by a double‐blinded peer‐review process. Furthermore, journal editors must recognize the consequences of implicit bias and think critically about how to address it. Rather than focusing exclusively on the novelty of methods in submissions, they should also consider each paper more holistically in terms of what is known about the particular species. For example, a paper reporting levels of genetic variation in a tropical species may use older methods, such as RAPD markers, but it could also be the first such study ever in that species. Therefore, despite lacking methodological novelty, the study could have value to the broader research community based on the new knowledge that it has generated. Unfortunately, journals may not be willing to embrace this approach due to their dependence on journal impact factor rankings (but see Berenbaum, 2019); however, this could change with development of other models that shift importance from journals to individual articles or researchers, such as the *h*‐index. Finally, publishers must think critically about how the fees they charge can impair access and prevent international researchers from contributing equally to their journals. Resources such as Research4Life (https://www.research4life.org) are also being developed to provide access to journals and to defray article publication charges for authors from eligible institutions and countries. Taken all together, these are the first steps needed to develop global equity in the plant sciences.

## What is *APPS* doing to address this disparity?

*Applications in Plant Sciences* (*APPS*) began in 2013 as a publication outlet for *all* authors throughout the plant sciences and has continued in this tradition to publish novel tools and methods. The journal is committed to publishing all types of methods (regardless of cost) provided that the method itself or the application is original. Over the past few years, the editors and staff have been carefully considering how to more effectively engage researchers from all over the world, as demonstrated in the following ways:

- **Encourage submissions from all countries:** Over the past year, submissions were received from the United States, China, Australia, and France but also from 19 other countries, including Pakistan, Mexico, Nigeria, Peru, and Kenya. We are now reviewing our author guidelines to ensure they are clear for authors for whom English is not their first language.
- **Reaching out to international authors:** Through our partnership with Wiley, we are expanding our efforts to reach out to authors through workshops in other countries, such as at the Congreso Latinoamericano de Botánica in Quito, Ecuador, in 2018, and the Congreso Mexicano de Botánica in Aguascalientes, Mexico, in 2019. We also offer waivers and discounts (https://authorservices.wiley.com/open‐research/open‐access/for‐authors/waivers‐and‐discounts.html) on publication charges for authors from low‐ and middle‐income countries.
- **Recruiting international reviewers:** Over the past three years, we have depended on the expertise of reviewers from 50 counties. While most reviewers have originated from the United States, United Kingdom, Canada, Germany, and China, researchers from countries such as Ecuador, Costa Rica, Vietnam, and Tunisia have also contributed their expertise as reviewers.
- **Open access papers:** All readers with internet access can readily access for free all articles in the journal as soon as they are published, as the journal is open access with articles available in HTML format and downloadable as PDF files.
- **Diversifying our editorial board to represent our global audience:** We have purposely expanded our Editorial Board and our Reviewing Editor Board to reflect the countries of origin, race/ethnicity, and gender of our authors and readers. Our editorial boards now represent 12 countries in addition to the United States: Argentina, Brazil, China, Germany, India, Italy, Korea, Mexico, Spain, Sri Lanka, Taiwan, and Venezuela.
- **Personalized review process with a sensitivity to international authors with English as an additional language:** All new submissions are quickly screened to ensure first that the scientific content is appropriate for the journal, and then that the English is of sufficient quality to facilitate review. Editors also work constructively with all authors throughout the review process and ultimately the production process to ensure that the scientific content of each paper is easily understandable by all readers.
- **Emphasis on low‐cost methods:** A special issue published in April 2020 titled “Conducting Botanical Research with Limited Resources: Low‐Cost Methods in the Plant Sciences” included 12 articles presenting effective methods that can be used by any researcher. Due to the high level of interest in this subject, a follow‐up special issue on the same topic is currently in the early planning stages.

​

Many plant biologists strive to become effective and productive researchers, developing solutions to complex problems or questions, contributing to past discoveries, and inspiring generations to come. One common misconception is that anyone can succeed if they have a strong work ethic, patience and determination, technical ability, and intellectual prowess. The reality is far from this, given that accessibility to resources is also a key component as demonstrated here. If we are to progress as a discipline, we will need the contributions of *all* researchers, regardless of where they are located around the globe. This requires that our profession reflect upon whether we are truly the equitable society we strive to be and work to build a more inclusive profession.
